# *Cucumber mosaic virus* coat protein induces the development of chlorotic symptoms through interacting with the chloroplast ferredoxin I protein

**DOI:** 10.1038/s41598-018-19525-5

**Published:** 2018-01-19

**Authors:** Yanhong Qiu, Yongjiang Zhang, Chaonan Wang, Rong Lei, Yupin Wu, Xinshi Li, Shuifang Zhu

**Affiliations:** 1 Chinese Academy of Inspection and Quarantine, Beijing, 100176 China; 2 China Agricultural University, Beijing, 100129 China

## Abstract

*Cucumber mosaic virus* (CMV) infection could induce mosaic symptoms on a wide-range of host plants. However, there is still limited information regarding the molecular mechanism underlying the development of the symptoms. In this study, the coat protein (CP) was confirmed as the symptom determinant by exchanging the CP between a chlorosis inducing CMV-M strain and a green-mosaic inducing CMV-Q strain. A yeast two-hybrid analysis and bimolecular fluorescence complementation revealed that the chloroplast ferredoxin I (Fd I) protein interacted with the CP of CMV-M both *in vitro* and *in vivo*, but not with the CP of CMV-Q. The severity of chlorosis was directly related to the expression of Fd1, that was down-regulated in CMV-M but not in CMV-Q. Moreover, the silencing of Fd I induced chlorosis symptoms that were similar to those elicited by CMV-M. Subsequent analyses indicated that the CP of CMV-M interacted with the precursor of Fd I in the cytoplasm and disrupted the transport of Fd I into chloroplasts, leading to the suppression of Fd I functions during a viral infection. Collectively, our findings accentuate that the interaction between the CP of CMV and Fd I is the primary determinant for the induction of chlorosis in tobacco.

## Introduction

Plant viruses generally induce mosaic symptoms on infected hosts. The chloroplast is a common target of plant viruses, and are closely related to the expression of mosaic symptoms^1,2^. Viruses affect chloroplasts via several ways^1,3^; they can repress the transcription of chloroplast-related genes^4–7^ and inhibit the translation of chloroplast -related proteins^8–11^, resulting in deformation and disruption chloroplast^12–14^. Viral factors also affect the function of some chloroplast proteins^3,15–17^. For example, the disease-specific protein of *Rice stripe virus* reportedly recruits the chloroplast oxygen-evolving complex protein, PsbP, into the cytoplasm by interacting with it^14^. Whereas, the HC-Pro proteinase of *Potato virus Y* was observed to interact with the chloroplast division-related factor NtMinD to inhibit the formation of NtMinD homodimers, thereby interfering with chloroplast division^18^. Furthermore, the P3 protein encoded by *Shallot yellow stripe virus* was revealed to directly disrupt ribulose-1,5-bisphosphate carboxylase/oxygenase activities in the infected plant host^19^.

*Cucumber mosaic virus* (CMV) is a member of the genus *Cucumovirus* in the family *Bromoviridae*^20^. This virus can infect more than 1200 plant species, causing significant economic losses in diverse crops of agricultural importance^21^. The CMV genome consists of three positive-sense RNA strands, encoding five proteins^22^. The 1a and 2a proteins are encoded by RNA1 and RNA2, respectively, and are obligatory for viral replication^22,23^. The 2b protein is encoded by a subgenomic RNA (RNA4A) from RNA2, and functions as a viral suppressor of RNA silencing^24^. RNA3 is dicistronic: The protein encoded by the 5′ open reading frame (ORF) is the designated movement protein, while the CP encoded by the 3′ ORF is translated from a subgenomic RNA4 synthesized de novo from RNA3 minus-strand progeny^22,25,26^.

The CMV strains are broadly divided into two subgroups (subgroup I and II) based on the nucleotide sequence homology^26^. CMV strains in the same subgroup, but not between the subgroups, share a high degree of sequence similarity^22,26^. Another feature that distinguishes CMV strains of the subgroup I and II is the severity of the symptom phenotype^26^. For example, CMV-Q belonging to subgroup I induce mild green mosaic phenotype^27^. By contrast, CMV strains M and Price 6 belonging to subgroup II induces strong chlorotic phenotype^28^. Analysis of pseudorecombinants revealed that RNA3 being the genetic determinant for the induction of symptom phenotype^29,30^. Finer mutational analysis later identified that amino acid residue located at position 129 affects symptom expression^31–33^. A more recent study showed that chloroplast damage is closely related to the onset of mosaic symptoms in *Nicotiana tabacum* cv. Samsun plants infected with CMV^13^. However, it remains unclear how the CP of CMV interferes with host chloroplasts to induce a given symptom phenotype.

In this study, the CP of CMV was confirmed to be the determinant for mosaic symptom phenotype in tobacco plants. Additionally, a chloroplast protein, ferredoxin I (Fd I), was found to interact with the CP of CMV both *in vitro* and *in vivo*, and was directly associated with the development of chlorosis symptoms. Further analyses revealed that the interaction destabilized Fd I and disrupted its import into chloroplasts, resulting in the development of chlorosis symptoms on tobacco leaves.

## Results

### Viral CP is the determinant of mosaic symptoms in the host

**Figure 1:**
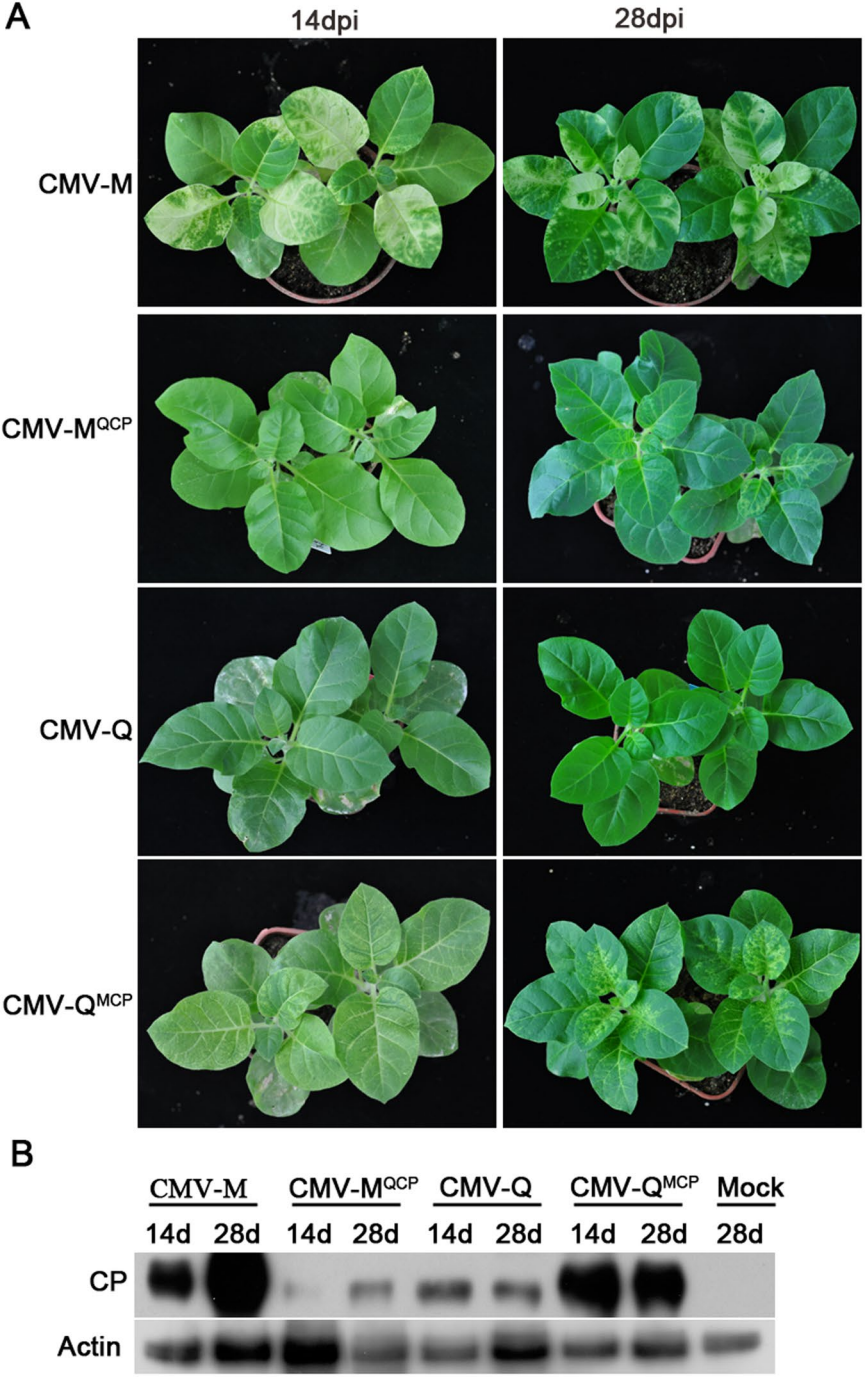
Symptoms induced by two CMV strains at 14 and 28 days post-inoculation (dpi). (**A**) The M strain of CMV (CMV-M) and recombinant virus, CMV-Q^MCP^, induced chlorosis symptoms on *Nicotiana tabacum* cv. Samsun plants. The Q strain of CMV (CMV-Q) did not induce symptoms on tobacco plants, while an infection by the recombinant virus, CMV-M^QCP^, resulted in a lack of symptoms at 14 dpi and mild green mosaic symptoms at 28 dpi. (**B**) The accumulation of CP in the top systemic leaves were detected by western blot analysis with an anti-CP monoclonal antibody. Plant actin was used as the control.

We used infectious CMV cDNA clones to investigate whether the viral CP regulated the development of mosaic symptoms. We exchanged the CP-encoding regions between two CMV strains that induced different symptoms. The M strain of CMV (CMV-M), from subgroup I, induced severe chlorosis symptoms on *Nicotiana tabacum* cv. Samsun plants, while the Q strain of CMV (CMV-Q), from subgroup II, caused no symptoms or mild green mosaic symptoms (Fig. 1A). When the CP-coding regions of CMV-M was replaced with that of CMV-Q, the recombinant virus, CMV-M^QCP^, did not cause any obvious symptoms at 14 days post-inoculation (dpi). However, infected plants started to exhibit mild green mosaic symptoms at 28 dpi (Fig. 1A). Plants inoculated with the reciprocal recombinant virus CMV-Q^MCP^ developed chlorosis symptoms at 14 dpi, and the symptoms were more severe by 28 dpi (Fig. 1A). The accumulation of CP in the top systemic leaves was assessed with an anti-CP monoclonal antibody. The level of virus accumulation differed among plants infected by CMV-M, CMV-Q, and their recombinant viruses (Fig. 1B). These results confirmed previous observations^13,31–33^ that the CP is determinant for mosaic phenotype.

### Chloroplast ferredoxin I interacts with the CP of CMV-M, but not the CP of CMV-Q

A yeast two-hybrid (YTH) assay was used to detect host proteins that interact with the CP of CMV-M. The CP-encoding ORF was inserted into pGBKT7 to produce the pGBK-MCP as a bait plasmid. A cDNA library for *N. tabacum* leaves was constructed using the pGADT7-Rec vector. Approximately 1.08 × 10^7^ independent cDNA library clones were obtained and used to transform yeast AH109 cells along with pGBK-MCP. A BLAST analysis against NCBI database sequences revealed that three clones had sequences that were almost identical to that of the gene encoding Fd I (GenBank accession No. AY552781), with only five nucleotides at the 5′ end of the gene being different.

**Figure 2:**
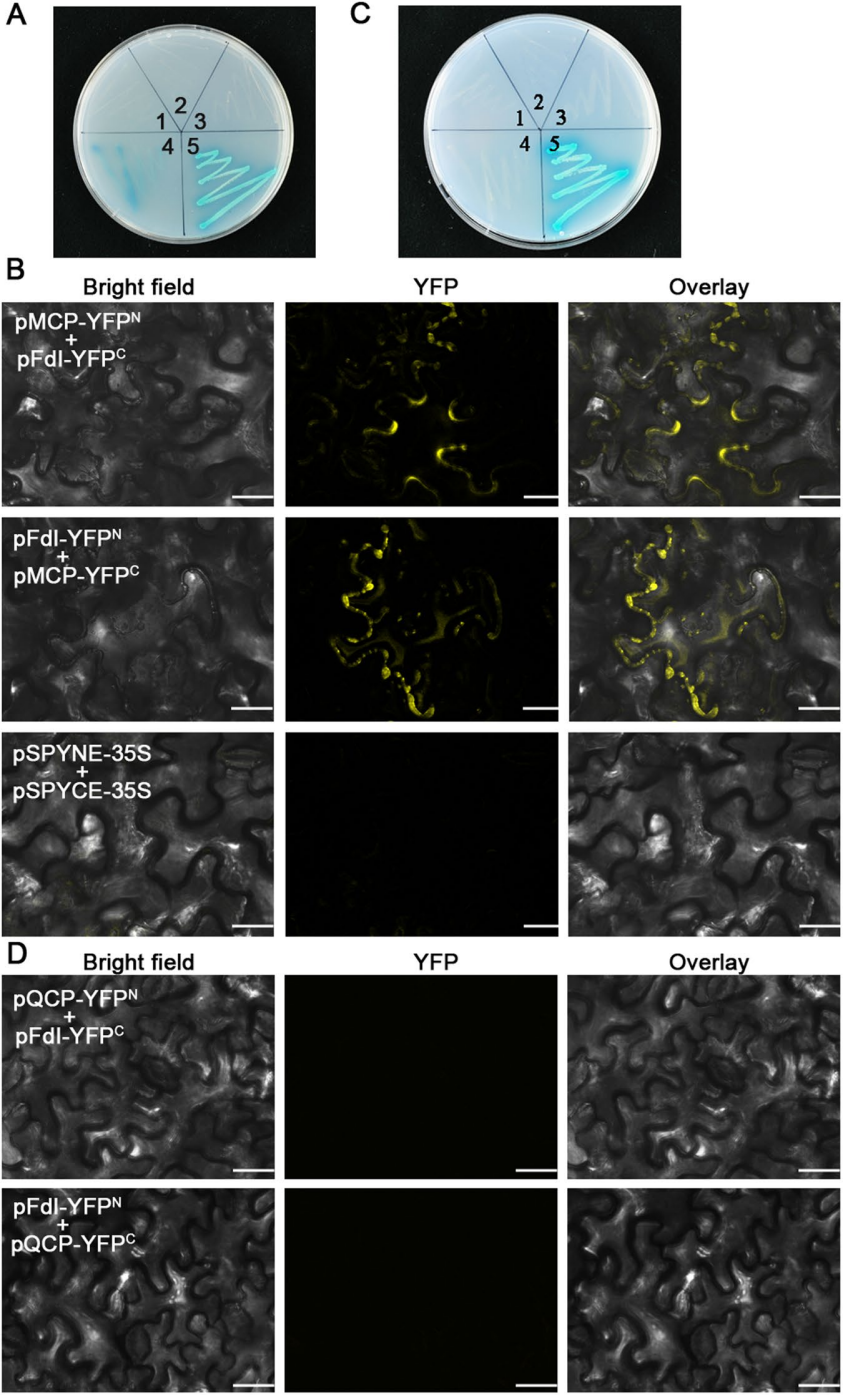
Interaction between ferredoxin I (Fd I) and the CP. (**A**) A yeast two-hybrid system was used to analyse the interaction between Fd I and the CP of CMV-M. Transformed *Saccharomyces cerevisiae* AH109 cells were cultured on SD/−Ade/−His/−Leu/−Trp/X-α-Gal medium. 1, pGADT7-RecT/pGBKT7-Lam (negative control); 2, pGADT7/pGBK-MCP; 3, pGAD-FdI/pGBKT7; 4, pGAD-FdI/pGBK-MCP; 5, pGADT7-RecT/pGBKT7-53 (positive control). (**B**) Bimolecular fluorescence complementation (BiFC) analysis to verify the interaction between Fd I and the CP of CMV-M in *Nicotiana benthamiana* epidermal cells. Two combinations of BiFC plasmids (pMCP-YFP^N^/pFdI-YFP^C^ and pFdI-YFP^N^/pMCP-YFP^C^) were inserted into plant cells with *Agrobacterium tumefaciens*, and YFP fluorescence was detected at 3 days post-agroinfiltration. The pSPYNE-35S/pSPYCE-35S plasmids were used as controls. Scale bars = 25 µm. (**C**) Yeast two-hybrid system to test the interaction between Fd I and the CP of CMV-Q. 1, pGADT7-RecT/pGBKT7-Lam (negative control); 2, pGADT7/pGBK-QCP; 3, pGAD-FdI/pGBKT7; 4, pGAD-FdI/pGBK-QCP; 5, pGADT7-RecT/pGBKT7-53 (positive control). (**D**) BiFC to verify the interaction between Fd I and the CP of CMV-Q in *N. benthamiana* epidermal cells.

The full-length Fd I cDNA sequence was cloned and inserted into pGADT7 to generate the pGAD-FdI plasmid, which was used to co-transform cells along with pGBK-MCP. The pGADT7-RecT/pGBKT7-53 plasmid combination was used as the positive control, while pGADT7-RecT/pGBKT7-Lam, pGADT7/pGBK-MCP, and pGAD-FdI/pGBKT7 served as negative controls. The transformants were cultured on SD/−Ade/−His/−Leu/−Trp medium and further verified by growing on SD/−Ade/−His/−Leu/−Trp/X-α-Gal medium. The AH109 cells transformed with pGAD-FdI/pGBK-MCP and pGADT7-RecT/pGBKT7-53 grew well on SD/−Ade/−His/−Leu/−Trp/X-α-Gal medium, while transformants harbouring pGADT7-RecT/pGBKT7-Lam, pGADT7/pGBK-MCP, or pGAD-FdI/pGBKT7 did not (Fig. 2A). These results indicated that Fd I interacts with the CP of CMV-M *in vitro*.

Bimolecular fluorescence complementation (BiFC) enables the direct visualization of complex protein interactions under natural conditions^34–36^. Plasmids for the production of the CP of CMV-M fused to the N-terminal of yellow fluorescent protein (YFP) and Fd I fused to the C-terminal of YFP or the reverse (pMCP-YFP^N^/pFdI-YFP^C^ and pFdI-YFP^N^/pMCP-YFP^C^) were inserted into *Agrobacterium tumefaciens* strain EHA105 cells, which were infiltrated into *Nicotiana benthamiana* epidermal cells. The pSPYNE-35S/pSPYCE-35S plasmids served as the negative control. After 2–3 days, cytoplasmic YFP signals were observed in plants carrying pMCP-YFP^N^/pFdI-YFP^C^ or pFdI-YFP^N^/pMCP-YFP^C^, but not in control plants (Fig. 2B). These results indicated that the CP of CMV-M interacts with Fd I in plant cells.

To investigate whether Fd I contributes to the variability in the expression of symptoms, we analyzed the interaction between Fd I and the CP of CMV-Q. The YTH and BiFC results indicated that Fd I was unable to interact with the CP of CMV-Q in yeast and plant cells (Fig. 2C and D). These findings implied that a specific interaction between Fd I and the CP of CMV-M affects the development of chlorosis symptoms.

### Ferredoxin I expression was affected by CMV-M infection, but not by CMV-Q infection

**Figure 3:**
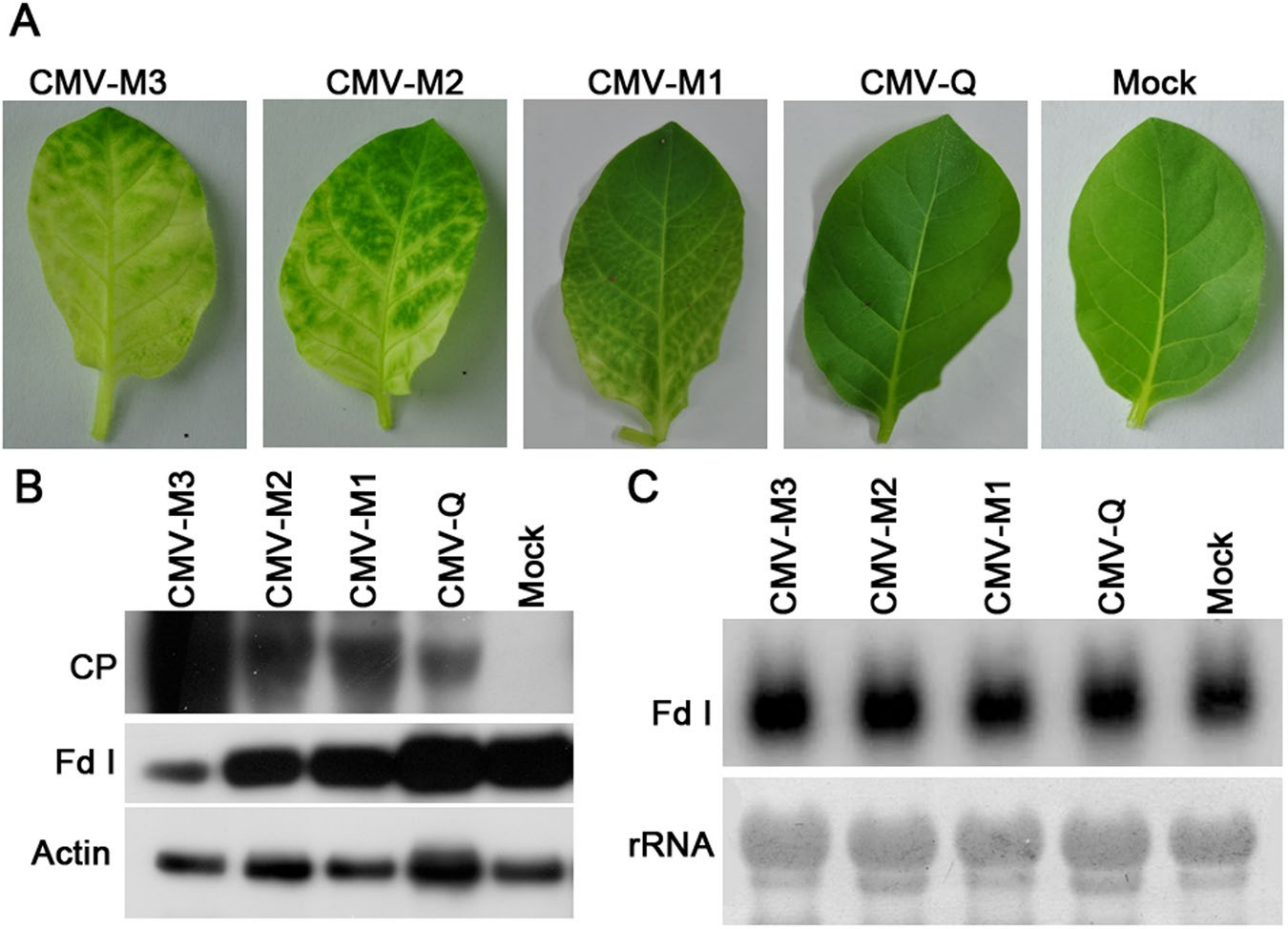
Detection of Fd I levels following an infection by CMV. (**A**) Leaves exhibiting severe chlorosis symptoms, mild chlorosis symptoms, or vein-clearing symptoms from CMV-M–infected *Nicotiana tabacum* cv. Samsun plants, and asymptomatic leaves from tobacco plants infected by CMV-Q and mock-inoculated plants. (**B**) Western blots using anti-CP monoclonal and anti-Fd I polyclonal antibodies to analyse CMV CP and Fd I in the abovementioned samples, with actin as a control. (**C**) Northern blot conducted with 10 µg total RNA to examine the accumulation of Fd I transcription using a specific probe corresponding to position 75–435 of Fd I mRNA. The loading control comprised 25 S rRNA.

To verify the roles of Fd I during symptom development, we monitored its abundance after viral infection. We collected the following three sets of leaf samples from CMV-M-infected *N. tabacum*: the first set of systemic leaves (CMV-M1) with vein-clearing symptoms, the second set of systemic leaves (CMV-M2) with mild chlorosis symptoms, and the third set consisted of asymptomatic leaves from CMV-Q–infected tobacco plants (Fig. 3A). The CP and Fd I contents were evaluated by western blot analysis with an anti-CP monoclonal antibody and an anti-Fd I polyclonal antibody, respectively. Plant actin was used as the internal control. The CP abundance increased as the chlorosis symptoms became more severe (CMV-M1 < CMV-M2 < CMV-M3). However, the abundance of Fd I clearly decreased during CMV-M infection, especially in leaves with severe chlorosis symptoms (CMV-M3). There was no clear decrease in Fd I contents in CMV-Q–infected samples relative to the level in mock-inoculated samples (Fig. 3B), suggesting that stability of Fd I was affected by CMV-M, but not by CMV-Q.

Viral infection has been shown to affect the expression levels of chloroplast-related genes^4,5,37–39^. A northern blot analysis was conducted to determine whether the decreased Fd I levels were associated with decreased Fd I mRNA content. Approximately 10 µg total RNA from each of the above-mentioned samples was subjected to agarose gel electrophoresis. The Fd I mRNA contents were similar in all five samples (Fig. 3C), implying that Fd I transcription was unaffected by the viral infection. Thus, the decrease in the abundance of Fd I may be due to its interaction with the CP of CMV-M.

### Decreased ferredoxin I levels induced chlorosis symptoms

**Figure 4:**
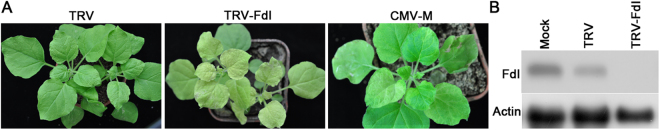
Symptoms induced by eliminating Fd I production. (**A**) Chlorosis symptoms on plants in which Fd I was silenced with a TRV-based virus-induced gene silencing system. The symptoms were similar to those induced by CMV-M on *Nicotiana benthamiana* plants. Additionally, there were no obvious symptoms on plants carrying the empty TRV vector. (**B**) Accumulation of Fd I in mock-, TRV-, and TRV-FdI–inoculated samples, as determined by western blot analysis, with plant actin as a control.

To determine whether the down-regulation of Fd I is directly related to the development of chlorosis symptoms, Fd I was silenced using a *Tobacco rattle virus* (TRV)-based virus-induced gene silencing (VIGS) system^40^. At 21 dpi, chlorosis symptoms were observed on *N. benthamiana* plants injected with TRV-FdI, similar to those induced by CMV-M infection (Fig. 4A). No obvious symptoms were detected on TRV-infected plants (Fig. 4A). A western blot analysis confirmed that the Fd I abundance had decreased in TRV-FdI–injected plants (Fig. 4B), which was likely directly related to the development of chlorosis symptoms.

### Viral infection inhibited transport of ferredoxin I into chloroplasts

**Figure 5:**
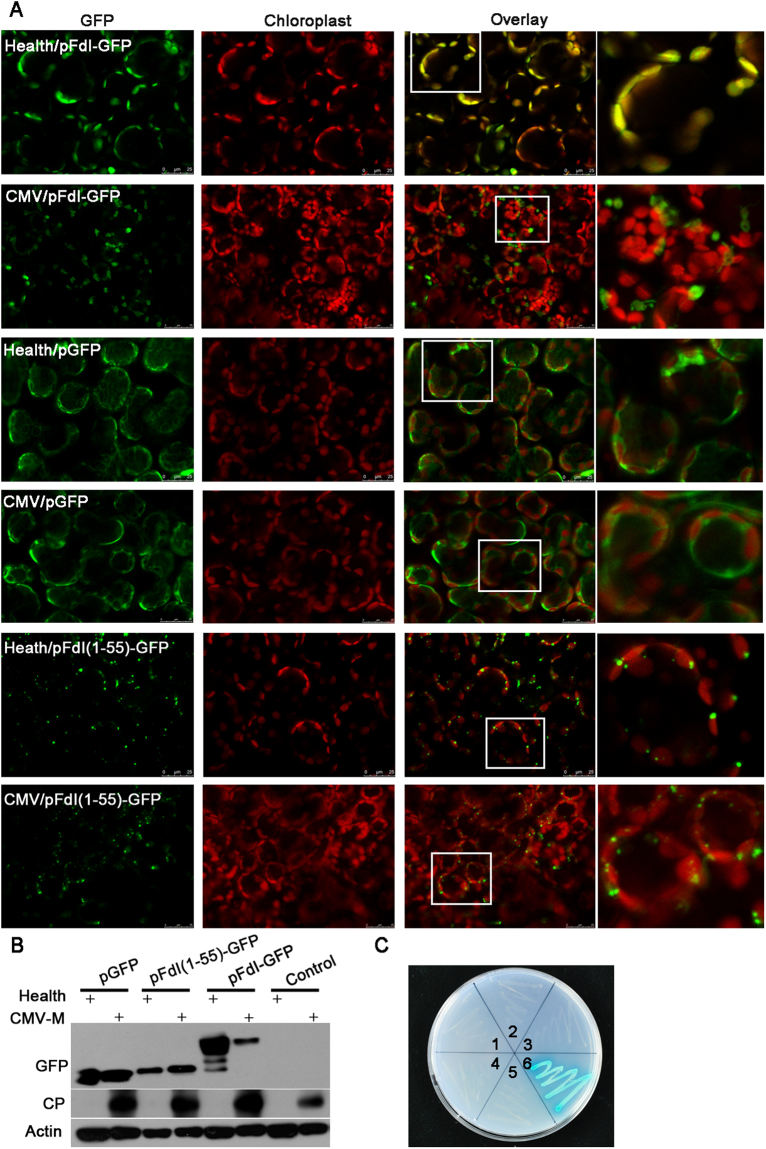
Subcellular production and localization of Fd I in CMV-M–infected or healthy *Nicotiana benthamiana* plants. (**A**) The Fd I-GFP fusion protein mainly localized in the chloroplasts and cytoplasm of healthy and CMV-M–infected plants, respectively. Whereas, GFP alone was mainly localized in the cytoplasm in healthy and CMV-M–infected hosts. Residues 1–55 at the N-terminal of Fd I formed a transit peptide targeting GFP into chloroplasts in both healthy and CMV-M–infected plants. Scale bars = 25 µm. (**B**) Western blot analysis with an anti-GFP antibody to detect GFP alone (pGFP), GFP fused to Fd I (pFdI-GFP), and GFP fused to the Fd I transit peptide [pFdI(1–55)-GFP]. (**C**) In yeast cells, the CP of CMV-M did not interact with the Fd I transit peptide or mature Fd I. 1, pGADT7-RecT/pGBKT7-Lam (negative control); 2, pGAD-FdI (1–55)/pGBKT7; 3, pGAD-FdI (56–145)/pGBKT7; 4, pGAD-FdI (1–55)/pGBK-MCP; 5, pGAD-FdI (56–145)/pGBK-MCP; 6, pGADT7-RecT/pGBKT7-53 (positive control).

Fd I is encoded by the nuclear genome. The precursor of Fd I is produced in the cytoplasm and then imported into chloroplasts^41^. To investigate whether the transport of Fd I is influenced by the viral infection, the Fd I ORF was inserted into the pGFP vector, which contains the ORF of the gene encoding the green fluorescent protein (GFP). The pFdI-GFP plasmid was infiltrated into both healthy and CMV-M-infected *N. benthamiana* plants with *A. tumefaciens*. The empty pGFP vector was used as the control. When pFdI-GFP was transferred into healthy plants, the fluorescence of the Fd I-GFP fusion protein was observed in chloroplasts. However, when pFdI-GFP was injected into the chlorotic leaves of CMV-M–infected plants, most of the Fd I-GFP fusion protein was observed in the cytoplasm instead of chloroplasts (Fig. 5A). The localization of GFP alone was not affected by CMV-M infection, as GFP fluorescence was observed in the cytoplasm in both healthy and CMV-M–infected plants (Fig. 5A). We also investigated the subcellular localization of the CP of CMV-M, and observed that CP-GFP fusion protein was localized in the cytoplasm (Supplementary Fig. S1). These findings indicated that the CP of CMV-M interfered with the import of Fd I into chloroplasts.

The import of Fd I into chloroplasts is mediated by a transit peptide (TP) that is removed inside chloroplasts to release the mature form of Fd I^42^. To determine whether the function of the Fd I TP is inhibited by the infection of CMV-M, we first identified the TP by generating constructs for the production of fusion proteins in which different parts of the Fd I N-terminal were fused to GFP. When GFP was linked to residues 1–55 of Fd I [pFdI(1–55)-GFP], GFP fluorescence was detected in chloroplasts (Fig. 5A), indicating that residues 1–55 at the N-terminus of Fd I were sufficient to target the protein to chloroplasts. When the pFdI(1–55)-GFP plasmid was infiltrated into healthy or CMV-M–infected plants, the Fd I TP-GFP fusion proteins was localized in the chloroplasts (Fig. 5A), confirming that the function of the Fd I TP was unaffected by CMV-M.

Western blots were used to detect GFP or GFP-tagged fusion proteins in healthy and CMV-M–infected *N. benthamiana* plants after the *A. tumefaciens*-mediated infiltration. The accumulation of the Fd I-GFP fusion proteins was much lower in CMV-M–infected *N. benthamiana* plants than in healthy plants, while the accumulation of GFP alone or the Fd I TP-GFP fusion proteins were similar in CMV-M–infected and healthy *N. benthamiana* plants. These results confirmed that the Fd I production was influenced by CMV-M.

To investigate whether CMV-M interacts with the Fd I TP, individual constructs containing sequences encoding either the Fd I TP (residues 1–55) or mature Fd I (residues 56–145) were cloned into pGADT7, generating the pGAD-FdI (1–55) and pGAD-FdI (56–145) plasmids, respectively. When these plasmids were used in a YTH assay with pGBK-MCP, the CP of CMV-M did not interact with the Fd I TP or the mature form of Fd I (Fig. 5C). This explained why CMV-M only interfered with the function and stability of the Fd I precursor, and not the Fd I TP.

## Discussion

In this study, we revealed that the chloroplast ptrotein, Fd I, interacted with the CP of CMV-M but not with that of CMV-Q. We also observed that the interaction influenced Fd I expresion and function to induce the formation of mosaic symptoms. The chloroplast is oftern targted by plant viruses and usually undergoes structural and functional changes during viral infection^1–3^. The CMV infection could induce mosaic symptoms, with the CP as an important determinant^31–33^. However, no chloroplast proteins had previously been confirmed to be associate with the CP. In this study, we confirmed that the chloroplasts protein Fd I can interact with the CP of CMV-M, but not the CP of CMV-Q. Subsequent analyses indicated that Fd I was associated with the expression of chlorotic symptoms, as the Fd I abundance decreased following an infection by CMV-M, but not by CMV-Q. Additionally, the chlorosis symptoms induced by the silencing of Fd I were the same as those resulting from an infection by CMV-M.

An earlier study concluded that Fd I interacts with the CP of *Tomato mosaic virus* to regulate chlorosis symptom development, although how the interaction induces the formation of the symptoms was not determined^43^. Researchers speculated that Fd I influences the transport of the viral CP into chloroplasts because the accumulation of the CP of *Tobacco mosaic virus* in chloroplasts increased when Fd I was silenced^44^. However, the results of our analyses suggest that Fd I has different functions during viral infections. We observed that the CMV-M infection interfered with the import of Fd I into chloroplasts, as most of the Fd I remained in the cytoplasm. Moreover, the CP of CMV-M localized in the cytoplasm, where it interacted with Fd I. Furthermore, the CP of CMV-M interacted with the precursor of Fd I synthesized in the cytoplasm, but not with the Fd I TP or the mature form of Fd I localized in chloroplasts. Thus, we concluded that the CP of CMV-M specifically interacts with Fd I to influence its function.

We also observed that Fd I levels decreased in response to an infection by CMV-M. Researchers previously suggested that the CP may influence the chloroplast-to-nucleus retrograde signals to repress the transcription of chloroplast-related genes^13^. However, our northern blot analyses indicated that the Fd I transcript levels were unaffected by the CMV infection. These results imply that the decrease in Fd I levels was related to the interaction with the CP of CMV-M. Consequently, we speculate that Fd I may fail to fold properly because of the interaction, thereby preventing it from being efficiently imported by chloroplasts. The accumulation of unfolded or misfolded proteins will inhibit normal cellular functions. Thus, the structurally unstable or abnormal Fd I may be selectively degraded by cell proteases machineries^45–48^.

**Figure 6:**
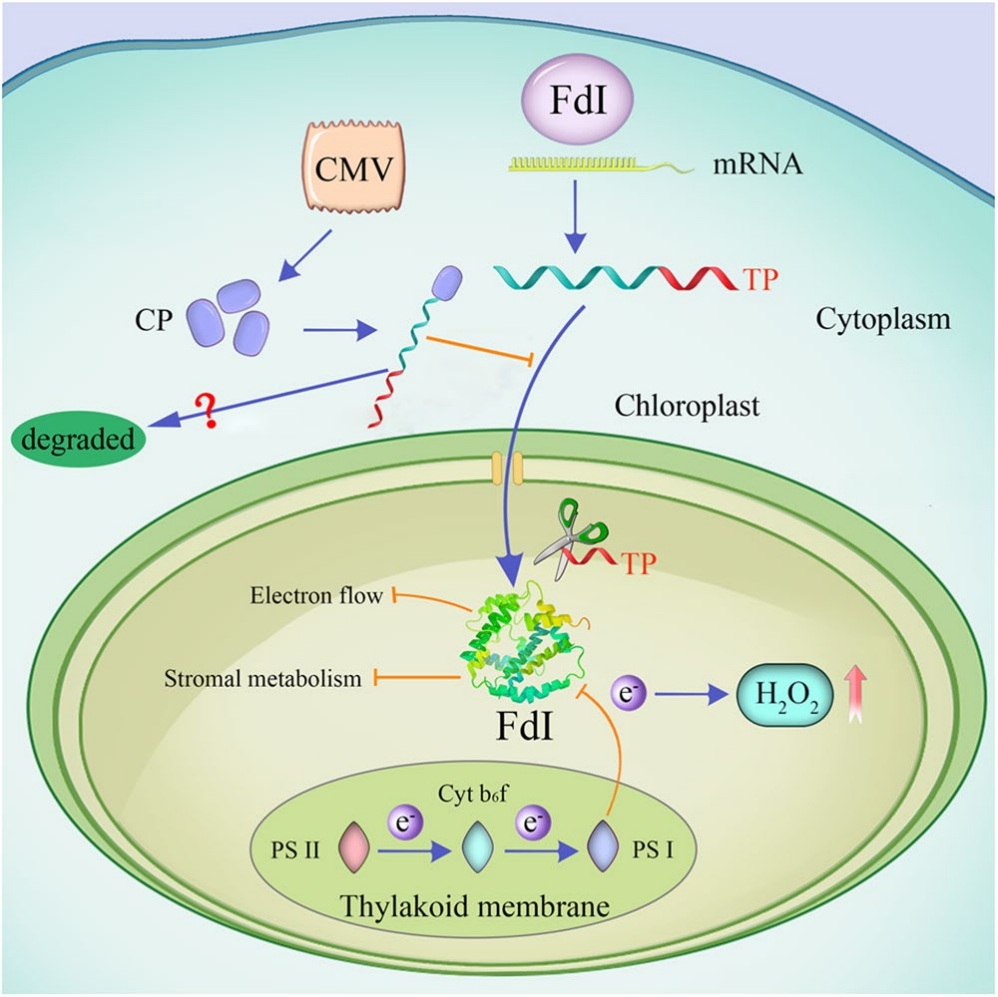
Model for Fd I functions during viral infections. After it is synthesized in the cytoplasm, the Fd I precursor is imported into chloroplasts because of the transit peptide (TP), which is removed inside the chloroplasts. Our data indicate that the CP of CMV interacts with the Fd I precursor to inhibit the import process. Decreases in Fd I production following an infection by CMV will need to be further verified. Because Fd I localizes on the stromal side of chloroplasts and transfers electrons from photosystem I (PS I) to various pathways, decreases in Fd I abundance will disrupt the electrons transport chain and influence Fd I-dependent metabolic activities. This will ultimately lead to an increase in H_2_O_2_ levels and inhibited chloroplast function.

Because Fd I is an indispensable part of photosystem I and donates electrons to various pathways^41^, a decrease in Fd I content alters the distribution of electrons^49^ (Fig. 6). The disrupted electrons transport in the plant host due to an infection by CMV stimulates the accumulation of H_2_O_2_ in chloroplasts^50^. The considerable oxidative stress imposed by H_2_O_2_ might be toxic to chloroplasts^44,51^. Moreover, many enzymes receive electrons from Fd I, implying a decrease in Fd I content affects the metabolic activities occurring in the chloroplast (Fig. 6), including the biosynthesis of chlorophyll and phytochrome^41^. Therefore, the abnormal production and translocation of Fd I resulting from an infection by CMV-M is responsible for the formation of chlorosis symptoms along with disrupted chloroplast structure and function^51^. However, the detailed mechanism by which Fd I is repressed *via* its interaction with the CP requires further research.

In addition to the CP, the 2b protein of CMV is also involved in the development of mosaic symptoms^26^. An infection by a 2b protein-deficient CMV-M mutant induces relatively weak chlorosis symptoms and a low viral accumulation in *N. tabacum* plants^52^. This suggests the 2b protein can enhance the accumulation of viruses by inhibiting several steps of the RNA silencing process^53^, but does not directly induce the development of mosaic symptoms^13,52,54^. By exchanging the CP between CMV-M and CMV-Q, we confirmed that the CP is a determinant of mosaic symptom development. Furthermore, the amino acid residue at position 129 of the CP influences the formation of symptoms on host plants. Modifying this amino acid was suggested to affect the three-dimensional structure of the CP^55^, viral assembly^32^, or viral movement^56,57^, but the effects on CP functions have not been fully characterized. Moreover, whether this amino acid participates in the interaction between the CP of CMV-M and Fd I will need to be investigated further.

In summary, the results presented herein indicate the CP of CMV-M interacts with Fd I and disrupts its synthesis and function, leading to the development of chlorosis symptoms on tobacco plants.

## Materials and Methods

### Plasmid construction

All vectors used in this study are listed in Table 1. Details of the primers used for cloning are provided in Supplementary Information Table S1.

**Table 1:** Details regarding the plasmids used in this study.

Plasmids	Primer	Instruction
pM3^QCP^	M-5UTR-F/M-5UTR-R, QCP-F/QCP-R, M-3UTR-F/M-3UTR-R	CP mutants and based on viral infectious cDNA clones.
pQ3^MCP^	Q-5UTR-F/M-5UTR-R, MCP-F/MCP-R, Q-3UTR-F/Q-3UTR-R	
pGBK-MCP	BD-MCP-F/BD-MCP-R	For YTH work and based on vector pGBKT7 and pGADT7 (Clontech).
PGBK-QCP	BD-QCP-F/BD-QCP-R	
pGAD-FdI	AD-FdI-F/AD-FdI-R	
pGAD-FdI(1–55)	AD-FdI-F/AD-FdI-55R	
pGAD-FdI(56–145)	AD-FdI-56F/AD-FdI-R	
pMCP-YFP^N^	Bi-MCP-F/Bi-MCP-R	For BiFC assay and based on vector pSPYNE-35S and pSPYCE-35S^36^.
pMCP-YFP^C^		
pQCP-YFP^N^	Bi-QCP-F/Bi-QCP-R	
pQCP-YFP^C^		
pFdI-YFP^N^	Bi-FdI-F/Bi-FdI-R	
pFdI-YFP^C^		
pFdI-GFP	Sub-FdI-F/Sub-FdI-R	For subcellular localization assay and based on modified vector pCAMBIA1300.
pFdI(1–55)-GFP	Sub-FdI-F/Sub-FdI-55R	
pTRV-FdI	TRV-FdI-F/TRV-FdI-R	For VIGS assay and based on TRV mediated VIGS system^40^.

### Plant growth and viral inoculation

*Nicotiana tabacum* cv. Samsun and *N. benthamiana* plants were grown in a glasshouse at 24 °C under a 16-h light/8-h dark photoperiod. The infectious CMV-M cDNA clones (i.e., pM1, pM2, and pM3) under the control of the T7 promoter were constructed in our laboratory. The infectious CMV-Q clones (i.e., pQ1, pQ2, and pQ3) were a gift from the Shouwei Ding Laboratory (University of California, Riverside, CA, USA)^58^. The CP-encoding ORF was exchanged between CMV-M and CMV-Q, resulting in two plasmids (i.e., pM3^QCP^ and pQ3^MCP^). Transcripts for the viral infectious clones pM1/pM2/pM3 (CMV-M), pM1/pM2/pM3^QCP^ (CMV-M^QCP^), pQ1/pQ2/pQ3 (CMV-Q), and pQ1/pQ2/pQ3^MCP^ (CMV-Q^MCP^) were obtained using a mMESSAGE mMACHINE^®^ Kit (Ambion), and were used to inoculate the top two fully expanded leaves of *N. tabacum* plants at the four-leaf stage.

### Yeast two-hybrid screening

A cDNA library for *N. tabacum* leaves was constructed using the Matchmaker™ Library Construction & Screening Kit (Clontech) according to the manufacturer’s instructions. The library was screened with a bait plasmid in co-transformed *Saccharomyces cerevisiae* strain AH109 cells. The yeast cells were then spread on synthetic dropout (SD) medium lacking adenine, histidine, leucine, and tryptophan (SD/−Ade/−His/−Leu/−Trp) to identify putative interacting proteins. To verify protein interactions, clones growing well on SD/−Ade/−His/−Leu/−Trp medium were used to inoculate into the same medium supplemented with X-α-Gal (SD/−Ade/−His/−Leu/−Trp/X-α-Gal). Plasmids were extracted from positive clones and sequenced for further functional analyses.

### Bimolecular fluorescence complementation analysis

We used the pSPYNE-35S and pSPYCE-35S BiFC vectors, which enabled the expressio of the N- and C-termini of YFP (YFP^N^ and YFP^C^, respectively)^36^. Using a double-digestion method, the ORFs of the CP genes from CMV-M and CMV-Q were cloned into pSPYNE-35S and pSPYCE-35S to produce the pMCP-YFP^N^, pMCP-YFP^C^, pQCP-YFP^N^, and pQCP-YFP^C^ plasmids. The Fd I ORF was also cloned into pSPYNE-35S and pSPYCE-35S, generating the pFdI-YFP^N^ and pFdI-YFP^C^ plasmids. The following plasmid pairs were used to transform *A. tumefaciens* strain EHA105 cells, which were grown in medium until the cultures reached the exponential growth phase: pMCP-YFP^N^/pFdI-YFP^C^, pFdI-YFP^N^/pMCP-YFP^C^, pQCP-YFP^N^/pFdI-YFP^C^, and pFdI-YFP^N^/pQCP-YFP^C^. We used needles to infiltrate the leaves of healthy *N. benthamiana* plants with the collected cultures. Leaves were collected after 48 or 72 h and observed under an SP5 confocal laser scanning microscope (Leica Microsystems). The excitation and emission wavelengths for YFP were 514 nm and 520–550 nm, respectively.

### Subcellular localization in plant cells

To study the subcellular localization of the target protein, the pCAMBIA1300 transient expression vector was modified to carry the ORF of the gene encoding GFP (pGFP). The Fd I ORF and an fragments encoding the N-terminal (corresponding to residues 1–55) were inserted into the pGFP vector to generate the pFdI-GFP and pFdI(1–55)-GFP plasmids, respectively. These constructs were incorporated into healthy and CMV-M–infected *N. benthamiana* plants using *A. tumefaciens* strain EHA105. To locate the GFP-tagged fusion proteins, GFP fluorescence and the auto-fluorescence of chloroplasts were induced by a laser (488 nm), and captured at emission wavelengths of 500–530 nm and 650–700 nm, respectively.

### Virus-induced gene silencing

The VIGS system is commonly used to study the function of plant genes^40^. We applied a TRV-based VIGS system, which used modified infected clones to transfer the DNA fragments that targeted homologous genes^40^. A sequence corresponding to nucleotide position 63–433 of tobacco Fd I mRNA was amplified and inserted into the pTRV2 vector using restriction enzyme digestions to produce the pTRV-FdI plasmid. This plasmid was then inserted into *A. tumefaciens* strain EHA105 cells together with pTRV1 (TRV-FdI). The empty pTRV1/pTRV2 vector were used as the control (TRV). Cultures were then injected into *N. benthamiana* plants at the five-leaf stage, with *N. benthamiana* plants infected with CMV-M served as positive controls. Plants were photographed using a digital camera (Nikon, Tokyo, Japan) at 21 dpi.

### Western blot analyses

Plant leaves were immediately ground in liquid nitrogen and mixed with 2× loading buffer for sodium dodecyl sulfate polyacrylamide gel electrophoresis. Proteins were then transferred to a polyvinylidene difluoride (PVDF) membrane (GE Healthcare) using a semi-dry transfer method. The PVDF membrane was blocked with 5% milk for 1 h at room temperature. After an overnight incubation with the primary antibody at 4 °C, the membrane was treated with a horseradish peroxidase-labelled secondary antibody (Sigma–Aldrich). Signals were visualized by exposing the PVDF membrane to an x-ray film after adding Pierce™ ECL Western Blotting Substrate (Thermo Scientific). The virus was detected with an anti-CP monoclonal antibody, while GFP was detected with an anti-GFP monoclonal antibody (Cell Signaling Technology). Plant actin was used as the internal control, and was detected with a polyclonal antibody (Sigma–Aldrich).

### Northern blot hybridization

Total RNA was extracted from plant tissue using the TRIzol reagent (Invitrogen). The extracted RNA was separated on a 1.2% agarose gel containing formaldehyde, transferred to a nylon N^+^-membrane (GE Healthcare), and cross-linked with ultraviolet light. The membrane was stained with a methylene blue solution (0.5 N NaAc, pH5.2 and 0.04% methylene blue) and rinsed with distilled water to produce clearly visible bands, which were used as the loading control. The DNA fragments corresponding to position 75–435 of the Fd I mRNA sequence were labeled with [α-^32^P]-dCTP using the Rediprime II DNA Labeling System (GE Healthcare). Signals were visualized on exposed film.

## Electronic supplementary material

Supporting Information
