# Supplementary material for: Cucumber mosaic virus coat protein induces the development of chlorotic symptoms through interacting with the chloroplast ferredoxin I protein
Source: Sci Rep. 2018 Jan 19;8:1205. doi: 10.1038/s41598-018-19525-5 (PMC5775247; doi:10.1038/s41598-018-19525-5)

***Cucumber mosaic virus* coat protein induces the development of chlorotic symptoms through interacting with the chloroplast ferredoxin I protein**

Yanhong Qiu[1], Yongjiang Zhang[1], Chaonan Wang[1, 2], Rong Lei[1], Yupin Wu[*1], Xinshi Li[*1] & Shuifang Zhu[*1]

[1]Chinese Academy of Inspection and Quarantine, Beijing 100176, China. [2]China Agricultural University, Beijing 100129, China. Correspondence and requests for materials should be addressed to Y.P.W (email: wu yuping@caiqtest.com) or X.S.L (email: lixinshi2017@126.com) or S.F.Z. (email: zhusf@caiq.gov.cn).

Supplementary materials

Supplementary Table

Table S1 Details regarding the primers used in this study.

Supplementary Figure

Figure S1 The subcellular localization of CMV CP. GFP fused to CP mainly localized in cytoplasm when *Agrobacterium*- infiltrated into *Nicotiana benthamiana* plants. Scale bars = 25 μm.

Table S1 Details regarding the primers used in this study.

Primer	Sequences	Restriction Enzyme
M-5UTR-F	GGATCCTAATACGACTCACTATAGGTAATCTTACCACTGTG	*Bam*HI/*Pst*I
M-5UTR-R	ATTTGTCCATGACTCGACTCAATTC	
QCP-F	ATTGAGTCGAGTCATGGACAAATCTGG	
QCP-R	CTGGAACACGGAACTAAGTCGGG	
M-3UTR-F	TGAGTCGAGTCATGGACAAATCTGG	
M-3UTR-R	CTGCAGTGGTCTCCTTTTAGAG	
Q-5UTR-F	CTGCAGTAATACGACTCACTATAGGGTAATCTTACCAC	*Pst*I/*Bam*HI
Q-5UTR-R	GATTTGTCCATAGGCACACTGAGAC	
MCP-F	GTCTCAGTGTGCCTATGGACAAATC	
MCP-R	AACACACGGATCAGACTGGGAGC	
Q-3UTR-F	GTGCTCCCAGTCTGATCCGTGTGTTTACCGG	
Q-3UTR-R	AACGGATCCTGGTCTCCTTATGGAGAACCTGTGG	
BD-MCP-F	CGGATCCGTATGGACAAATCTGAATC	*Bam*HI/*Pst*I
BD-MCP-R	ACTGCAGATCAGACTGGGAGCACTC	
AD-FdI-F	AAAGAATTCATGGCCAGTATTTCAGGTAC	*Eco*RI/*Bam*HI
AD-FdI-R	AGGGATCCTTAGCCAGTGAGCTCC	
AD-FdI-55R	AAAGGATCCTTAAATAAGCTTCACTTTGTAACTG	
AD-FdI-56F	AGGAATTCATGACACCAGAGGGAACAGTTGAG	
Bi-MCP-F	AACGGATCCATGGACAAATCTGAATC	*Bam*HI/ *Kpn*I
Bi-MCP-R	AAAGGTACCTCAGACTGGGAGCACTCTAG	
Bi-QCP-F	AAAGGATCCATGGACAAATCTGGATCTCC	*Bam*HI/ *Kpn*I
Bi-QCP-R	AAAGGTACCAGTCGGGAGCATCCGTGAG	
Bi-FdI-F	AACGGATCCATGGCCAGTATTTCAGG	*Bam*HI/ *Xho*I
Bi-FdI-R	AAACTCGAGGCCAGTGAGCTCCTCC	
Sub-FdI-F	AACTGCAGATGGCCAGTATTTCAGG	*Pst*I/*Sal*I
Sub-FdI-55R	AAAGTCGACAAATAAGCTTCACTTTGTAACTG	
Sub-FdI-R	AGTCGACATGCCAGTGAGCTCCTC	
Sub-CP-F	AACTGCAGATGGACAAATCTGAATC	*Pst*I/*Spe*I
Sub-CP-R	AAACTAGTGACTGGGAGCACTCTAG	
TRV-FdI-F	AAGAATTCCCTGAAAGCCATACC	*Eco*RI/
TRV-FdI-R	AAGGATCCAGACTGTGGGTAAGC	*Bam*HI

Figure S1

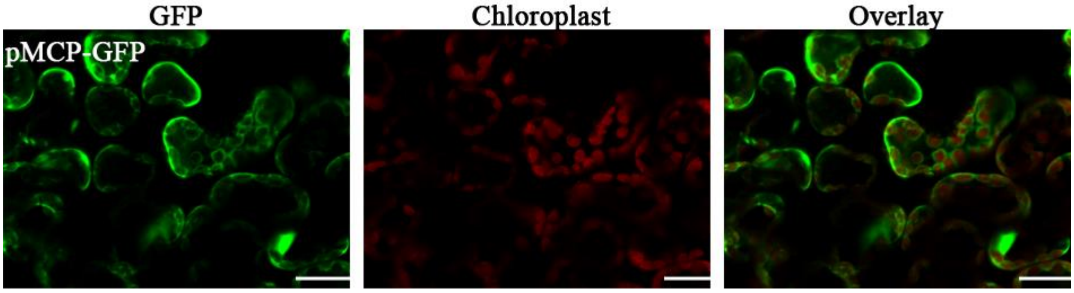

Supplement: Supplementary file 1 — Supporting Information [file 41598_2018_19525_MOESM1_ESM.pdf]
